# Gout treatment and comorbidities: a retrospective cohort study in a large US managed care population

**DOI:** 10.1186/1471-2474-12-103

**Published:** 2011-05-20

**Authors:** Paola Primatesta, Estel Plana, Dietrich Rothenbacher

**Affiliations:** 1 Global Clinical Epidemiology, Novartis Pharma AG, CH-4056 Basel, Switzerland; 2 Global Clinical Epidemiology, Novartis Farmacéutica SA, Barcelona, Spain; 3 Institute for Epidemiology and Medical Biometry, University of Ulm, Ulm, Germany

## Abstract

**Background:**

Gout prevalence increased in recent years to become one of the most common causes of inflammatory arthritis in most industrialised countries. Comorbidities may affect the disease severity and treatment patterns. We describe the main characteristics of gout patients, gout-related treatment patterns and prevalent comorbidities in a managed care population.

**Methods:**

From the large US PharMetrics Patient-Centric Database, patients aged 20-89 with at least 2 claims for a diagnosis of gout (ICD9 274.xx) and related prescriptions between January 1, 1996 and December 31, 2008 were included. Gout flares were ascertained during follow-up. Sex-specific multivariable Poisson regression models were used to assess factors associated with number of flares.

**Results:**

177,637 gout patients were included (mean age 55.2 years; men 75.6%). Overall, more than half (58.1%) had any of the considered comorbidities; hypertension (36.1%), dyslipidemia (27.0%) and diabetes (15.1%) being the most common. Nonselective NSAIDs were the most commonly dispensed (in 38.7% of patients). Notably, 39% of patients did not receive any prescription medication for gout. Patients with comorbidities were significantly more likely to receive anti-gout prescriptions. During an acute episode the prescription of NSAIDs and colchicine increased; and 29.9% of patients received allopurinol. The risk of flares was associated with cardiometabolic comorbidities and older age in women (highest at age 60-69), while in men it decreased by age. Women with these conditions were 60% more likely to have flares (incidence rate ratio, IRR 1.60;1.48-1.74), while men were 10% (IRR 1.10; 1.06-1.13) more likely.

**Conclusions:**

Comorbidities affected gout treatment patterns and the occurrence and frequency of acute attacks. Cardiometabolic comorbidities, common in this patients' population, were associated with an increased risk of flares.

## Background

Gout prevalence has been increasing in recent years and it is currently one of the most common causes of inflammatory arthritis in most industrialised countries [1]. The increase in the disease prevalence has been linked to increased lifespan, dietary habits, increase in obesity and alcohol consumption; and use of drugs that may raise serum urate levels [1,2]. The most important single risk factor for developing gout is a raised serum uric acid level. Gout results from the deposition of urate crystals in a joint, leading to an acute inflammatory response. One acute attack may be the only manifestation, or acute inflammatory arthritis episodes (flares), often monoarticular, may recur. If untreated this may lead to chronic gouty arthropathy and deposition of urate crystals in soft tissues, forming tophi.

Acute gout attacks are painful and potentially disabling, needing immediate treatment. The optimal therapy is directed at controlling pain and inflammation [3,4]. Drug therapy for gout has become an important part of the therapeutic approach to the disease, which includes lifestyle modifications, and guidelines for its management are available internationally [5,6]. Current standards for first-line treatment for acute attacks include nonsteroidal anti-inflammatory drugs (NSAIDs), colchicine and corticosteroids. Urate-lowering treatment (ULT) (most commonly allopurinol) is usually recommended after the acute attack has resolved.

Despite growing understanding of the disease aetiology and risk factors involved, evidence is accumulating that part of the patient populations is not optimally treated and managed and continue to experience clinical manifestations of gout [7,8]. Moreover, suggested first-line therapies are often not well tolerated, and may be contraindicated in some patients due to coexistent medical problems, especially in the elderly, as the majority of these patients have pre-existing conditions such as diabetes, renal, cardiovascular or gastrointestinal diseases [9,2].

The objective of this study was to describe the main characteristics of a large population of patients with gout, their gout-related treatment patterns and prevalent comorbidities and to investigate the frequency and risk factors of flares during follow-up using a managed care population from the US.

## Methods

This retrospective cohort study was conducted in the PharMetrics Patient-Centric Database, an administrative claims database which contains medical and pharmacy claims for > 55 million patients from > 90 managed care health plans in the US. Access to the PharMetrics database requires a licence agreement and the data are provided deidentified. Tabulations for these data do not require ethical approval.

Records in the database are representative of the national commercially insured population on a variety of demographic variables, including age, gender, geographic distribution and plan type. The average member enrolment time is 2 years. All patients aged 20-89 years with at least 2 claims (either primary care, office visit or emergency room visit) for a diagnosis of gout (International Classification of Diseases 9th Edition (ICD9) 274.xx) between January 1, 1996 and December 31, 2008 were identified from the anonymized database. The index date was defined as the first occurrence of a gout claim during the study period. Patients were only included in the study if they had been continuously enrolled in the health plan for the period between 6 months before and one year after the index date.

Gout flares were ascertained during the follow-up period. Gout flares were defined as a claim reporting gout as defined above, together with at least one of the following within one week: a) treatment with NSAIDs, colchicine, oral corticosteroids, or adrenocorticotropic hormone (ACTH); b) intraarticular aspiration or injection (Current Procedural Terminology (CPT) codes 206.00, 206.05 or 206.10). Given that an untreated gout flare can last up to a few weeks, each gout flare was estimated to last 30 days and a new flare episode was counted only if it had occurred beyond the 30-day period. The results related to number of flares included a 12 months follow-up period starting 30 days after index date.

Gout medication treatment patterns were identified from the pharmacy claims. Use of NSAIDs (selective/non-selective), colchicine, corticosteroids (oral or injections), probenecid, and allopurinol was ascertained during a period of 12 months following the index date; and within a week following a flare, to capture drug prescription following an acute episode.

Comorbidities were identified based on the ICD9 codes, during the 6 months before the index date, for claims with a diagnosis of congestive heart failure, ischemic heart disease, hypertension, diabetes, cerebrovascular disease, dyslipidemia, chronic renal failure, gastrointestinal disease, haematological disease, myopathy, osteoporosis.

Data were described using simple descriptive statistics. Sex-specific multivariable Poisson regression models were used to assess factors associated with number of flares. The covariates considered in the multivariable regression model included established risk factors, i.e. age and history of comorbidities (cardiometabolic, gastrointestinal, haematological, myopathy, osteoporosis). All statistical analyses were carried out using SAS (version 9.2).

## Results

Table 1 shows the main characteristics of the 177,637 gout patients included in the study (12 patients were excluded because they had a previous diagnosis of gout within 6 months of the index data). Mean age was 55.2 years (SD 13.1). Men represented the majority of patients (75.6%). Overall, more than half (58.1%) had any of the considered comorbidities; hypertension (36.1%), dyslipidemia (27.0%), diabetes (15.1%) and ischemic heart disease (10.2%) being the most common.

**Table 1:** Demographic characteristics of gout patients (N = 177,637)

	N	%
Male gender	134,333	75.6%
Age in categories		
20-49	59,371	33.4%
50-59	54,612	30.7%
60-69	37,016	20.8%
70-79	19,790	11.1%
80-89	6,848	3.9%
Comorbidities:		
Cardiometabolic	91,203	51.3%
Congestive Heart Failure	7,467	4.2%
Ischemic Heart Disease	18,066	10.2%
Hypertension	64,094	36.1%
Conduction and rhythm disorder	12,034	6.8%
Cerebrovascular disease	4,934	2.8%
Peripheral arterial disease	6,271	3.5%
Chronic renal impairment	5,718	3.2%
Dyslipidemia	47,960	27.0%
Diabetes	26,818	15.1%
Gastrointestinal	14,224	8.0%
Gastrointestinal	11,836	6.7%
Hepatic disorders	2,718	1.5%
Pancreatic disorders	501	0.3%
Haematological (Anemia)	3,829	2.2%
Myopathy	159	0.1%
Arthritis	25,516	14.4%
Osteoporosis	5,044	2.8%
*Any comorbidity*	*103,281*	*58.1%*
Gout diagnosis recorded by:		
Primary care physician	56,512	31.8%
Rheumatologist	4,731	2.7%
Emergency visit	10,482	5.9%
Other or Unknown specialty	105,912	59.6%
Hospitalized in 12 months follow-up (at least once)	2,119	1.2%
Outpatient visit in 12 months follow-up (at least once)	65,665	37.0%
Number of flares in 12 months follow-up:		
0	158,268	89.1%
1	15,893	9.0%
2	2,790	1.6%
3 or more	686	0.4%

During the 12 months following the index date, 1.2% of patients were hospitalised at least once and 37.0% underwent an outpatient visit. In almost one third of patients (31.8%) the index visit was carried out at the primary care physician office; in only 2.7% it was conducted by a rheumatologist, while it was not known who made the diagnosis in almost 60% of the cases.

Overall 32,244 patients had at least 1 acute gout episode recorded in the database during the follow-up period (mean follow-up (SD) was 2.16 (1.45) years). According to the operational definition of flare used, in the 12 months of follow-up after the first occurrence of a gout claim (starting 30 days after index date) 11% of patients (N = 19,369) had experienced one or more flares.

Table 2 shows the gout related drugs utilization for the whole cohort and separately for men and women in the 12 month of follow-up. Nonselective NSAIDs were the most commonly dispensed (38.7% ever used them: 40.5% of men and 33.3% of women), followed by allopurinol (35.5%), corticosteroids (20.4%) and colchicine (18.3%) in men; and corticosteroids (22.7%), allopurinol (20.6%) and colchicine (11.8%) in women. Notably, 39% of patients (36.9% of men and 45.3% of women) did not receive any prescription medication for gout.

**Table 2:** Gout related treatment patterns overall and by presence of comorbidities

Gout-related medication	Ever use during 1 year after gout diagnosis										
	**All**		**No comorbidities** **(n = 74,356)**		**Cardiometabolic§** **(n = 64,385)**			**Diabetes** **(n = 26,818)**		**Chronic renal impairment** **(n = 5,718)**	
**ALL**	**n**	**%**	**n**	**%**	**n**	**%**	p-value*	**n**	**%**	**n**	**%**

NSAIDS	74,055	41.7%	32,701	44.0%	25,994	40.4%	0.022	9,592	35.8%	1,134	19.8%
selective	12,455	7.0%	4,102	5.5%	5,077	7.9%	< .0001	1,942	7.2%	219	3.8%
nonselective	68,826	38.7%	31,167	41.9%	23,765	36.9%	0.173	8,660	32.3%	1,001	17.5%
Colchicine	29,686	16.7%	11,595	15.6%	11,327	17.6%	< .0001	4,950	18.5%	1,559	27.3%
Corticosteroids	37,224	21.0%	14,182	19.1%	14,476	22.5%	< .0001	5,153	19.2%	1,617	28.3%
Probenecid	2,510	1.4%	1,108	1.5%	901	1.4%	0.249	331	1.2%	58	1.0%
Allopurinol	56,544	31.8%	23,215	31.2%	20,971	32.6%	< .0001	9,183	34.2%	2,512	43.9%
***No treatment***	***69,241***	***39.0%***	***29,202***	***39.3%***	***24,938***	***38.7%***	***< .0001***	***10,669***	***39.8%***	***2,171***	***38.0%***

**FEMALES**	N = 43,304		N = 12,387		N = 17,418			N = 9,381		N = 2,025	

NSAIDS	16,183	37.4%	4,641	37.5%	6,464	37.1%	< .0001	3,279	35.0%	368	18.2%
selective	3,815	8.8%	911	7.4%	1,643	9.4%	< .0001	769	8.2%	77	3.8%
nonselective	14,423	33.3%	4,255	34.4%	5,684	32.6%	0.000	2,899	30.9%	320	15.8%
Colchicine	5,096	11.8%	890	7.2%	2,312	13.3%	< .0001	1,558	16.6%	483	23.9%
Corticosteroids	9,831	22.7%	2,438	19.7%	4,213	24.2%	< .0001	1,954	20.8%	575	28.4%
Probenecid	441	1.0%	96	0.8%	195	1.1%	0.359	115	1.2%	27	1.3%
Allopurinol	8,905	20.6%	1,676	13.5%	4,006	23.0%	< .0001	2,692	28.7%	834	41.2%
*No treatment*	*19,637*	*45.3%*	*6,200*	*50.1%*	*7,693*	*44.2%*	*< .0001*	*3,991*	*42.5%*	*816*	*40.3%*

**MALES**	N = 134,333		N = 61,969		N = 46,967			N = 17,437		N = 3,693	

NSAIDS	57,872	43.1%	28,060	45.3%	19,530	41.6%	0.575	6,313	36.2%	766	20.7%
selective	8,640	6.4%	3,191	5.1%	3,434	7.3%	< .0001	1,173	6.7%	142	3.8%
nonselective	54,403	40.5%	26,912	43.4%	18,081	38.5%	0.012	5,761	33.0%	681	18.4%
Colchicine	24,590	18.3%	10,705	17.3%	9,015	19.2%	< .0001	3,392	19.5%	1,076	29.1%
Corticosteroids	27,393	20.4%	11,744	19.0%	10,263	21.9%	< .0001	3,199	18.3%	1,042	28.2%
Probenecid	2,069	1.5%	1,012	1.6%	706	1.5%	0.260	216	1.2%	31	0.8%
Allopurinol	47,639	35.5%	21,539	34.8%	16,965	36.1%	< .0001	6,491	37.2%	1,678	45.4%
*No treatment*	*49,604*	*36.9%*	*23,002*	*37.1%*	*17,245*	*36.7%*	*< .0001*	*6,678*	*38.3%*	*1,355*	*36.7%*

§ Cardiometabolic disease include congestive heart failure, ischaemic heart disease, hypertension, cerebrovascular disease, peripheral arterial disease, dyslipidemia.

* P-value from logistic model with drug as outcome associated to cardiometabolic comorbidities vs no comorbidities adjusting for age in categories (20-49, 50-59, 60-69, 70-79, 80-89).

Patients with cardiometabolic comorbidities were significantly more likely to be dispensed selective NSAIDs (7.9% vs 5.5%), colchicine (17.6% vs 15.6%), corticosteroids (22.5% vs 19.1%) and allopurinol (32.6% vs 31.2%) than gout patients with no comorbidites (all p < 0.0001 after adjusting for age). This was true for both genders. Patients with diabetes and cardiovascular comorbidities had similar patterns of anti-gout treatment prescriptions. Compared with those with no comorbidities, patients with renal impairment were less likely to be dispensed nonselective NSAIDs (17.5%), and more likely to receive allopurinol (43.9%), colchicine (27.3%) and corticosteroids (28.3%) (all p-values < 0.0001).

Table 3 shows the gout related drugs dispensed in the 7 days after any flare. During a flare the prescription of NSAIDs and colchicine increased. Almost a third (29.9%) of patients received allopurinol during an acute attack.

**Table 3:** Treatment patterns for treatment of flares occurring at any time during the study period

Gout drugs within 7 days of flare		
	(any flare) n = 32,244	
	n	%
NSAIDS	21,633	67.1%
selective	1,283	4.0%
nonselective	20,748	64.3%
Colchicine	12,847	39.8%
Corticosteroids	8,598	26.7%
Probenecid	420	1.3%
Allopurinol	9,644	29.9%
*No treatment*	*0*	*0.0%*

Patients with acute attacks were also more likely to receive treatment within 12 months following the index date, compared with the gout population who did not experience flares, as shown in Table 4. In particular, the corresponding numbers dispensed colchicine among patients with no, 1, 2, and 3 or more flares were 12.2%, 49.9%, 66.5% and 80% respectively. 27.9% of patients with no flares received allopurinol during the 12 months of follow-up, vs. 60.8%, 77.8% and 87.8% of patients with 1, 2, and 3 or more flares respectively. Patients with 3 or more flares were also more likely to be dispensed corticosteroids (70.6%) than those with 1 flare (45.7%) (Table 4).

**Table 4:** Treatment patterns: patients with no flares and with flares occurring during follow-up of 12 months (N = 19,369)

Ever during 12 months	No flares		Total (any flare)		1 flare		2 flares		3 flares	
after index date			(n = 19,369)		(n = 15,893)		(n = 2,790)		(n = 686)	
	n	%	n	%	n	%	n	%	n	%
NSAIDS	58325	36.9%	15730	81.2%	12821	80.7%	2338	83.8%	571	83.2%
selective	10110	6.4%	2345	12.1%	1795	11.3%	430	15.4%	120	17.5%
nonselective	53750	34.0%	15076	77.8%	12278	77.3%	2245	80.5%	553	80.6%
Colchicine	19353	12.2%	10333	53.3%	7929	49.9%	1855	66.5%	549	80.0%
Corticosteroids	28379	17.9%	8845	45.7%	6695	42.1%	1666	59.7%	484	70.6%
Probenecid	1781	1.1%	729	3.8%	503	3.2%	174	6.2%	52	7.6%
Allopurinol	44110	27.9%	12434	64.2%	9662	60.8%	2170	77.8%	602	87.8%
*No treatment*	*69241*	*43.7%*	*0*	*0.0%*	*0*	*0.0%*	*0*	*0.0%*	*0*	*0.0%*

Table 5 shows the results of the multivariable analysis. The risk of flares was associated with older age in women (highest at age 60-69), while in men it decreased by age. It was also positively associated with cardiometabolic comorbidities. Women with these conditions were 60% more likely to have flares (incidence rate ratio, IRR 1.60;1.48-1.74), while men were 10% more likely (IRR 1.10; 1.06-1.13). Gastrointestinal diseases were negatively associated with flares in women (IRR 0.86; 0.78-0.96) while a positive association was seen in men (IRR 1.07; 1.02-1.13). (Table 5).

**Table 5:** Poisson multivariate regression model for number of flares during follow-up of 12 months by sex

		Females (N = 43,304)			Males (N = 134,333)		
		IRR	95% CI	p-value	IRR	95% CI	p-value
Age	< 50	1.00	(ref.)		1.00	(ref.)	
	50 - 59	1.66	(1.49, 1.85)	< .0001	0.83	(0.80, 0.85)	< .0001
	60 - 69	1.93	(1.73, 2.16)	< .0001	0.76	(0.73, 0.79)	< .0001
	70 - 79	1.69	(1.51, 1.90)	< .0001	0.56	(0.53, 0.60)	< .0001
	80 - 89	1.31	(1.12, 1.52)	0.001	0.53	(0.48, 0.59)	< .0001
Comorbidities:							
Cardiometabolic*		1.60	(1.48, 1.74)	< .0001	1.10	(1.06, 1.13)	< .0001
Diabetes		1.48	(1.35, 1.63)	< .0001	0.97	(0.93, 1.02)	0.262
Gastrointestinal		0.86	(0.78, 0.96)	0.005	1.07	(1.02, 1.13)	0.011
Hematological		1.03	(0.88, 1.20)	0.720	0.94	(0.84, 1.06)	0.340
Myopathy		1.21	(0.50, 2.91)	0.670	0.84	(0.50, 1.42)	0.509
Osteoporosis		0.88	(0.78, 1.00)	0.044	1.11	(0.99, 1.25)	0.076

IRR = Incidence rate ratio.

* Cardiometabolic disease include congestive heart failure, ischaemic heart disease, hypertension, cerebrovascular disease, peripheral arterial disease, dyslipidemia.

When use of diuretics was entered in the model it showed a positive association with flares both in women (IRR 4.34; 4.03-4.68) and in men (IRR 1.9 1; 1.50-1.98) (data not shown).

## Discussion

This study, based on a US administrative claims database, examined prescription patterns, comorbidities and flares determinants in a very large population of gout patients. In line with current guidelines [5], NSAIDs and colchicine were the drugs most commonly prescribed during an acute attack. However our data suggest that somewhat suboptimal treatment and management may occur in clinical practice: almost a third of patients received ULT during an acute attack and 39% of patients did not receive any prescription medication for gout. Cardiometabolic comorbidities in particular were associated with gout treatment patterns and were also associated with a high burden of disease, as reflected by an increased risk of flares.

Gout treatment has been shown to be suboptimal in two studies from the US [10,11] and a study from the UK [12]. According to our data, gout treatment was relying mostly on traditional treatment means such as NSAIDs to control inflammation and pain. Corticosteroids and colchicine were dispensed in about a fifth of patients while ULT, recommended as the treatment to prevent further acute attacks, was dispensed in less than a third of all gout patients and probenecid was seldom dispensed. Probenecid use was also described as infrequent in a study based on US ambulatory care data [13]. Here the authors, basing their study on a national probability sample survey of physicians-patients encounters, showed a larger overall utilisation of allopurinol in patients with gout than in our study. Differences in estimates may be related to differences in data collection methods and patients characteristics (e.g. age and geographic location). For example, the age distribution of this patient population appeared to be younger compared to other studies. On average, only 12% of the PharMetrics population was aged 60-89. This may make the results of our study less generalizable to the gout population overall, however consistent with the age distribution in other health care plans [14].

NSAIDs have been reported as the drug of first choice to treat acute gout attacks in the US [2] and in other countries [12]. These drugs are, however, associated with gastrointestinal, renal and cardiovascular effects and it was not surprising that in our study patients with cardiometabolic comorbidities, and in particular those with chronic renal impairment, had the prescription of NSAIDs greatly reduced. It is notable that almost two fifths of patients did not receive any prescription within 12 months from the index date. These patients may have had fewer flares, i.e. less severe manifestation of the disease, may have had medication still at home, or been able to purchase over the counter medications to reduce their symptoms, since non-prescription medications would not be recorded in the database.

Given that hyperuricemia is the most important single risk factor for gout, the long-term management of hyperuricemia to prevent further attacks and complications of the disease is an important component of the therapeutic regimen. The use of ULT is recommended after the acute episode has subsided [5], however according to our data almost a third of patients received a prescription of allopurinol during an acute attack. An increased number of flares during the 12 months from the first occurrence of a gout claim seemed to have resulted in more aggressive treatment patterns. In particular, more than three times as many patients with 3 or more flares were dispensed corticosteroids or allopurinol compared with patients with no flares. This may reflect the fact that in patients with more severe disease (using number of flares as an indicator of disease severity) the available therapeutic regimen was not adequate to prevent further acute attacks, and the treating physician had to step up the treatment.

The association between cardiovascular disease and gout, including significant cardiovascular mortality risk, is well recognised [15-17]. EULAR guidelines therefore recommend screening for cardiovascular risk and treating comorbid conditions in gout patients [5]. Our study demonstrated that cardiometabolic comorbidities were frequent in this patient population. They impacted the prescription patterns, since these patients were more likely to be prescribed corticosteroids, colchicine, selective NSAIDs and allopurinol than patients with no comorbidities. The increased use of selective NSAIDs in patients with cardiometabolic comorbidities is somewhat counterintuitive given their potential cardiovascular risk (although data collection partly preceded recent cardiovascular concerns), even though the EULAR guidelines are still open on this issue [5]. Regarding the increased use of allopurinol in patients with cardiometabolic comorbidities, this may also be considered to go against clinical judgement, in particular considering its potential toxicity in patients with impaired renal function and the potential for drug interaction (e.g. warfarin). Whether allopurinol should be prescribed at reduced dosage in patients with impaired renal function remains however still controversial [5]. These patients were also at increased risk of flares. The use of diuretics is a recognised risk factor for gout. Although contraindicated [5], these medications are commonly prescribed in this patients' population, and this may increase their risk of flares.

Women with gastrointestinal diseases were at reduced risk of flares while in men the association was in the opposite direction. This observation has not been made before and should get further consideration in future studies.

Patients with renal impairment were less likely to be prescribed NSAIDs than gout patients without comorbidities, and more likely to receive allopurinol, colchicine and corticosteroids. It is recommended that these patients receive a lower dose of allopurinol given that they are at increased risk of toxic effects [14]. Providing adequate information to these patients regarding the risks and benefits at different dosages would be advisable.

Limitations of a study based on an administrative claims database need to be considered. Firstly, these data only apply to patients with a commercial insurance and these findings may therefore not be generalisable to gout patients without commercial insurance. Further, the diagnosis of gout was not confirmed using medical record review. To increase the sensitivity and specificity of the gout identification algorithm, a patient was only classified as a gout patient if two distinct medical encounters with a diagnosis of gout were present in the record. This would reduce the potential for including in the study patients without gout. Currently there is no validated epidemiological definition of gout flare, and although we followed the definition used by other authors (cfr e.g. ref [14]), based on clinical measurements, we expect that some errors of sensitivity (failure to identify true gout flares) and specificity (identification of subjects who did not experience gout flares) may be inherent in the definition. Specifically, some patients may, when starting ULT, have received NSAIDs, colchicine and, less commonly, steroids as prophylaxis against acute attacks and this may have lead to an overestimation of flares. However, by excluding those who commenced allopurinol treatment within 7 days before a flare the number of patients with flares would not have changed substantially (19,267 vs 19,369 observed).

It is also recognised that many patients with gout flares do not consult their physician [11], and may self-manage acute episodes. This is likely to be the case in the present study, given that the risk of gout flares reported over one year follow-up (10.9%) is lower than what other studies have shown [18]. It was also not possible to verify that the drugs included were dispensed for gout and to assess adherence to the prescribed treatment. Nevertheless, epidemiological studies such as the present one, which included a very large number of patients with gout, with a large US geographic distribution, can improve our understanding of management of gout in real world settings.

## Conclusions

In conclusion, results from the analysis of data from this large administrative claims database are supporting suggestions from other studies [10-12], i.e. that suboptimal treatment and management of gout may occur in clinical practice. In addition, cardiometabolic comorbidities, common in this patient population, were associated with a higher burden of disease, as reflected by an increased risk of flares.

## Competing interests

PP and EP are employees of Novartis Pharma.

DR was employee of Novartis Pharma until November 2010.

## Authors' contributions

PP conceived of the study, participated in its design and drafted the manuscript. She is responsible for the whole study. EP participated in the design of the study and performed the statistical analysis. DR participated in the design of the study and helped to draft the manuscript. All authors read and approved the final manuscript.

## Pre-publication history

The pre-publication history for this paper can be accessed here:

http://www.biomedcentral.com/1471-2474/12/103/prepub
